# Jones Tries It on

**Published:** 1905-07

**Authors:** 


JONES TRIES IT ON.—Jones, the sec.-editor of *The California State Journal of Medicine,* takes a fall out of the "Red Back" (nit)—and spells it with a little r-b. He tried it on with the *New York Medical Journal,* and got left. Said "its editorial pages have been bartered for coin," and had to take it back. (See "A Word of Apology," page 201, July number.) Criticising an editorial in the "Red Back" for May that he didn't like, Jones went out of his way to say, and said, "* * * a journal which refers editorially to itself as the red-back. One wonders what its editorial nickname for itself would be if the color of the binding were gray instead of red."

That's real funny—or silly, as you look at it. It is as if I should say, "Wonder what Jones' name would be if it were Smith? Or, what was it before it *was* Jones?" Smith would do as well; it is equally as distinctive. "*Which* Jones?" one asks. Well, any old Jones will do, the woods are full of 'em, and all Joneses look alike to me,—but "this here" Jones may be distinguished from ten thousand other Joneses by his contempt for English grammar,—as well as for being the editor of the California tentacle of the

Chicago Octopus. In speaking of the Octopus piling up money, he says: "With this excess amount good interest bearing securities are purchased for the benefit of *who* [sic] or what?" Yep; that's what "this here" Jones said. (Page 200, July number.)

